# When cooperation begets cooperation: the role of key individuals in galvanizing support

**DOI:** 10.1098/rstb.2015.0012

**Published:** 2015-12-05

**Authors:** Katherine McAuliffe, Richard Wrangham, Luke Glowacki, Andrew F. Russell

**Affiliations:** 1 Department of Psychology, Yale University, 2 Hillhouse Avenue, New Haven, CT 06520, USA; 2 Department of Human Evolutionary Biology, Harvard University, 11 Divinity Avenue, Cambridge, MA 02138, USA; 3 Department of Psychology, Boston College, Chestnut Hill, MA 02467, USA; 4 Program for Evolutionary Dynamics, Harvard University, 1 Brattle Square, Cambridge, MA 02138, USA; 5 Centre for Ecology and Conservation, College of Life and Environmental Sciences, University of Exeter, Treliever Road, Penryn, Cornwall TR10 9FE, UK

**Keywords:** biological market theory, coercion, cooperative matching, incomplete compensation, information asymmetry, shifting cost-benefit functions

## Abstract

Life abounds with examples of conspecifics actively cooperating to a common end, despite conflicts of interest being expected concerning how much each individual should contribute. Mathematical models typically find that such conflict can be resolved by partial-response strategies, leading investors to contribute relatively equitably. Using a case study approach, we show that such model expectations can be contradicted in at least four disparate contexts: (i) bi-parental care; (ii) cooperative breeding; (iii) cooperative hunting; and (iv) human cooperation. We highlight that: (a) marked variation in contributions is commonplace; and (b) individuals can often respond positively rather than negatively to the contributions of others. Existing models have surprisingly limited power in explaining these phenomena. Here, we propose that, although among-individual variation in cooperative contributions will be influenced by differential costs and benefits, there is likely to be a strong genetic or epigenetic component. We then suggest that selection can maintain high investors (*key individuals*) when their contributions promote support by increasing the benefits and/or reducing the costs for others. Our intentions are to raise awareness in—and provide testable hypotheses of—two of the most poorly understood, yet integral, questions regarding cooperative ventures: why do individuals vary in their contributions and when does cooperation beget cooperation?

## 1. Introduction

Fitness is enhanced by forwarding more gene copies to following generations than conspecifics from the same population [1,2]. Consequently, non-identical conspecifics are generally expected to compete rather than to cooperate [3]. Even when cooperation offers potential benefits, opportunities to free ride on the contributions of others are expected to be taken [2,4,5]. If some co-investors pay less and/or gain more than others, the resulting pay-off asymmetries can lead to defection in favour of non-cooperation [6–8]. Thus, even when cooperation among non-identical conspecifics is theoretically beneficial, there is a high potential for it to be unstable [3,9–11]. Yet, life abounds with examples of cooperation, and cooperation is implicated in several major evolutionary transitions, including the success of eukaryotes and complex societies [12]. Understanding how cooperation is stabilized despite its inherent difficulties is therefore an important problem.

**Figure 1.**
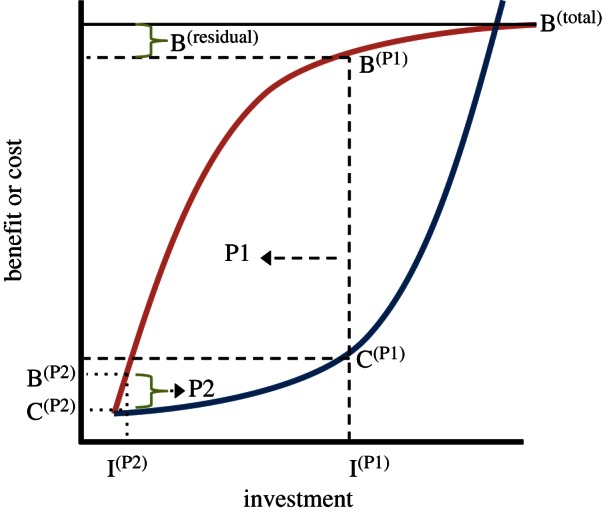
Stabilizing cooperation through partial responses. Theoretical treatments of cooperation have typically been conducted for bi-parental or simple cooperative care systems [15–18]. Consider the case where increasing investment (I) yields diminishing benefits (B, upper curve) and accelerating cost (C, lower curve). In the absence of co-investors, an individual (P1) is expected to invest in parameter space, where B^(P1)^–C^(P1)^ is maximal. Assuming this to be the case: imagine the arrival of a co-investor (P2), with a similar benefit and cost function to the original investor (P1). With a total benefit on offer of B^(total)^, without any change of investment by P1, P2 should only invest I^(P2)^ with benefit B^(P2)^ and cost C^(P2)^. Note that net B^(P2)^ is equal to B^(residual)^. Under this scenario, the relative fitness costs and benefits emerging from the two players' investments is highly asymmetric and the interaction, all else being equal, will be unstable. The optimal solution is for P1 to partially reduce its investment following the arrival of P2 (left-hand arrow), ‘forcing’ P2 to elevate its investment partially (right-hand arrow). Under partial compensation, contributors should invest similarly to B^(total)^, and any defection by one leads to reductions in fitness because the other members of the pair/group only partially compensate. (Online version in colour.)

Within an intra-specific context, cooperation can take one of two broad forms. In the simplest case, two or more individuals can invest in each other directly. Examples include gamete-trading, mutual grooming and sequential food sharing [5]. In such cases, cooperation is likely to be maintained by various forms of turn-taking strategy in which investment is minimized at each stage (e.g. reciprocal altruism, tit-for-tat or conditional strategies [4,10,13,14]). Alternatively, two or more individuals can invest in a common venture (i.e. a public good), with examples including joint investment in rearing and protecting offspring, domicile building or food gathering [5]. Most theory on optimal levels of co-investment between genetically non-identical individuals has been applied to bi-parental and cooperative care systems [15–18]. Such theory typically assumes that increasing investment is associated with diminishing benefit functions and accelerating cost functions, and makes two predictions (figure 1): (i) cooperation is maintained by partial-response strategies, i.e. increases in investment by one investor are met with fractional decreases by another (and *vice versa* for initial decreases); and (ii) because of partial responses, individuals should contribute relatively equitably to the public good. However, as we shall see below, violations of these theoretical predictions are sufficiently numerous to require explanation.

**Figure 2.**
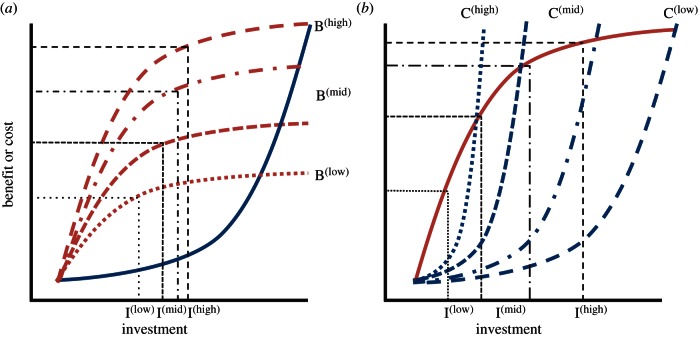
Selection on variable contributions through differential benefits and costs. (*a*) Variation driven by benefits: when all individuals have the same cost function (concave-up in this case, solid line), individuals gaining more benefit (e.g. high (dashed line) versus mid (dotted-dashed line) versus low (dotted line)) will be selected to invest at a higher level (I^(high)^ versus I^(mid)^ versus I^(low)^; lines as above) because the point of maximal difference between costs and benefits increases with increasing benefits. Differential benefit functions can arise for multiple reasons, including due to differences in relatedness to offspring [9,19], good versus poor circumstances [23] and/or differential benefits of contributing in the presence of an audience [20]. (*b*) Variation driven by costs: when all individuals have the same benefit function (e.g. concave-down, solid line), individuals with lower cost functions (e.g. low versus mid versus high; lines as for *a*) will be selected to invest at high levels (I^(high)^ versus I^(mid)^ versus I^(low)^; lines as above), in this case because the points of maximal differences between costs and benefits increase with declining costs. (Online version in colour.)

At least five hypotheses have been proposed to account for cases that do not meet the predictions of classic partial-response models; offering explanations for significant variation in individual contributions and/or opposing response strategies. First, individuals standing to gain more benefits (whether direct or indirect) from a cooperative venture might be expected to contribute more than those with less to gain ([2,9,19–22]; figure 2*a*). Second, those that can afford higher investment without concomitant reductions in benefits might be expected to contribute relatively more than individuals for which cooperating is more costly ([24–26]; figure 2*b*). Third, the best response can be to match the contributions of co-investors when they have more information on the state of the public good [27]. Fourth, individuals might be coerced into contributing more than is optimal based on benefit–cost analysis [28,29] or be selected to contribute more than expected due to competition in a biological market [30]. Finally, recent modelling has shown that individual contributions can be greater when benefit functions arising from increasing investment increase nonlinearly [21,31,32]. While these five hypotheses offer significant explanatory power in some cases, they appear to have limited utility in others.

This paper focuses on two findings that are surprising in light of traditional theory, and the potential link between them: (i) apparently stable cooperative ventures can be associated with substantial asymmetries in investment; and (ii) individuals can often react positively, not negatively, to increasing work rates of co-investors. We seek to account for individual variation in contributions by discussing the role of differential benefits and costs and—finding that these have limited explanatory power—suggest that genetic and/or epigenetic effects need to be considered as additional explanations. This conclusion raises the question of how (epi)genetic variation can be maintained. We suggest that one solution is positive matching of others' contributions. We therefore consider the generality of existing models to account for positive response rules, i.e. information asymmetries [27], coercion [29] and biological market theory [30]. Finally, we propose an additional hypothesis based on the role of key individuals in increasing the benefits and reducing the costs of contributions for others. In essence, we propose that the overall shape of individual benefit and cost functions can be modulated by the behaviour of others, and that key individuals can play a decisive role in this.

To illustrate the generality of our points, we use a hand-selected case study approach from a broad and significant literature base, including: (i) bi-parental care in birds; (ii) cooperative breeding in vertebrates and social insects; (iii) cooperative hunting; and (iv) cooperation in humans. Our intention is to stimulate future theoretical and empirical work in an essential yet relatively unexplored area of sociobiology.

## 2. Four case studies

### (a) Contributions to bi-parental care

In bi-parental care systems, unrelated mothers and fathers cooperate to rear young. In such systems, both members of the pair often, but not always, contribute similarly to offspring provisioning [33], and in many, mothers are the sole incubators and fathers the chief defenders [34,35]. A large theoretical and empirical research literature exists to explain variation in patterns of bi-parental care [35]. The key aspect that we wish to highlight here is that such systems have been used extensively to test how one member of the pair responds to manipulation of the provisioning contribution of the other. By and large, the expected pattern of incomplete compensation in response to partner manipulation is supported [33]. One notable exception has been recently documented by Hinde [36] and later Hinde & Kilner [37] in great tits (*Parus major*). In this species, both members of the breeding pair provision the offspring. When one member of the pair was experimentally subjected to increased begging signals, and hence a perception of increased brood demand, not only did they elevate their provisioning rate but so did their partner. Thus, partner contributions were positively (not negatively) associated. We discuss a similar case with cooperatively breeding long-tailed tits (*Aegithalos caudatus*) below. Regardless of whether such findings prove to be rare, they clearly defy the classic expectation of incomplete compensation [27].

### (b) Contributions in cooperative breeding systems

#### (i) Substantial variation and its consequences on others in cooperative vertebrates

In cooperative breeding systems, parental care is performed by parents as well as by non-breeding helpers. A major focus in studies of cooperatively breeding vertebrates has been to quantify and understand among-individual variation in contributions to rearing offspring. A universal feature emerging from such studies is that non-breeding helpers vary widely in how much they contribute to rearing the offspring of others, sometimes by up to two to three orders of magnitude [38–40]. For example, in meerkats (*Suricata suricatta*), one to two non-breeders can conduct up to 80% of all babysitting in a group [41]. In chestnut-crowned babblers (*Pomatostomus ruficeps*), non-breeders feed offspring at rates ranging from 0.1 to over 10 times per hour [42]. Although the probability that non-breeding group members contribute at all is commonly predicted by gender [38] or relatedness [43,44], as we shall see (§3), explaining the quantitative variation in contributions is surprisingly difficult.

One factor that is known to influence individual contributions to cooperative breeding is the number of helpers in a group [45], but the direction and strength of this relationship is not straightforward. Helper presence or increases in the number of helpers (rather than levels of investment *per se*) are known to be associated with complete reduction, partial reduction, no reduction and even increases in the investment of others [42,45–47]. This variety of responses highlights that theoretical expectations of partial-response rules are commonly violated [48]. Unfortunately, there has been a paucity of experiments aimed at understanding the substantial variation observed within groups, and those that have attempted to do so have highlighted the methodological difficulties. For example, Wright & Dingemanse [49] found that in Arabian babblers (*Turdoides squamiceps*), helpers that had not been supplementally fed fully reduced provisioning rates in response to increases by fed helpers, but noted that this could be due to nestlings becoming quickly satiated. McDonald *et al.* [50] found that temporarily (2-h) removing breeders from groups of bell minors (*Manorina melanophrys*) did not influence the average provisioning rate of those remaining, although it is unclear whether longer term removals may have had an effect or whether some responded while others did not. In the same species, McDonald *et al.* [51] reported positive responses to targeted playback-induced increases in provisioning rates of unrelated helpers and breeding males, suggesting positive matching. Together, these studies hint at intriguing complexity in how individuals respond to each other's investments in cooperation and highlight a pressing need to conduct further targeted experiments in conjunction with appropriate analytical techniques [52,53], in order to understand the stark among-individual variation in contributions to ‘public goods' in cooperative breeders.

#### (ii) Galvanizing effects in social ants

Although relatively few studies have considered quantitative variation in helper investment in social insects (but see [54]), there are some intriguing exceptions of relevance here. For example, in their classic book on ants, Hölldobler & Wilson [55] described a series of reports on a small numbers of workers (there termed elitists) that have been observed performing most of the work [55, p. 281]. The presence of such elitists can have profound effects on the activity of colonies. Möglich & Hölldobler [56] found that when colonies of the ants *Formica sanguinea* and *Camponotus sericeus* moved their nests, a small proportion of workers (in their colonies 11% and 6%, respectively) did much of the work. When these individuals were removed, the time taken in nest-moving was significantly extended. In their review of key individuals in insect societies, Robson & Traniello [57] classify key individuals like these workers as *organizers* of group labour. While Möglich and Hölldobler's observations suggest that this small group of workers is critical for mobilizing group activities, future work could provide firmer evidence of their role by including a control group in which the appropriate proportion of non-organizers is removed to ensure that differences in work rate are due specifically to the absence of key individuals as opposed to reduced group size.

To our knowledge, only one study has provided firm experimental evidence that key individuals can have a galvanizing effect on the work rates of lazier workers. In a study of carpenter ants (*Camponotus japonicus* var. *aterrimus* Emery), Chen [58] manipulated the social effects of hard-working individuals during cooperative nest-building. By pairing workers that had previously been shown to work at an intermediate rate with a ‘rapid worker’, Chen found that the intermediate worker worked harder (carried more material) and faster. Such individuals have since been classified as *catalysts* of group labour [57]. More recently, a spattering of further studies has investigated key individuals in insect societies [59,60], but to our knowledge no study since Chen's [58] has tested whether such individuals galvanize cooperation in others. Consequently, the mechanisms allowing for the spread of cooperation between individuals are currently unknown. We view this as a fruitful area for future inquiry.

### (c) Contribution to cooperative hunting: the case of chimpanzees (*Pan troglodytes*)

Male chimpanzees are opportunistic hunters of monkeys. Hunts normally result from a chance encounter between a party of chimpanzees on the ground and a group of monkeys (e.g. red colobus, *Procolobus* spp.) in the tree canopy. Red colobus groups include multiple adult males who provide aggressive and often effective defences against chimpanzee attackers. Hunts are more likely to occur when more male chimpanzees are in the party (e.g. rising from less than 5% with two males to approximately 40% with 10 males [61]). Hunting probability also increases when the costs of hunting are low (e.g. during times of high alternative plant food availability [62]) or when the chance of success is high (e.g. when escape routes for the monkeys are limited [63]). About 50% of hunts lead to at least one kill, with each kill normally being made by a single individual. Although prey can be stolen by a higher-ranking male, it is generally torn into sufficient number of pieces that many or all males, and often females and young, are able to eat some meat [61].

Gilby *et al.* [61] investigated the mechanism by which the presence of more adult males leads to a greater probability of hunting. Male chimpanzees at Kanyawara in Kibale National Park, Uganda, varied widely in their hunting rate. Two individuals in particular were much more likely to hunt than others and, critically, hunts rarely occurred in their absence. These ‘impact hunters' (here termed key individuals) were hypothesized to promote cooperative hunting by being the first to climb towards the monkeys, now evidenced by Gilby [64]. Such initiative is apparently costly for the performer because it attracts defensive mobbing and physical attacks by adult male monkeys. It may be beneficial for subsequent hunters, however, since when the defensive power of the monkeys is focused on the impact hunters, the chance of other hunters making a kill seems likely to rise [61].

### (d) Contribution to cooperative goals in humans

Humans often contribute differentially to a shared cooperative goal, whether within a family setting or in society at large. However, few studies have quantified this variation or attempted to explain it. We use data from public goods games, investment in the work environment and small-scale societal warfare to illustrate not only that humans vary in their level of contribution to a public good, but that increases in investment by one can galvanize increases by others.

#### (i) Public goods games

Experiments using public goods games consistently show substantial variation in the propensity of individuals to contribute to a public good [65,66]. For example, Kurzban & Houser [66] were able to classify public goods game players into three types: strong cooperators, strong free riders and conditional cooperators. They found that 25% of the players were strong cooperators who contributed the majority of their endowment to the public good, irrespective of what other players were contributing and their financial situation. More recently, Weber & Murnighan [67] confirmed that such consistent contributions by players occur spontaneously and demonstrated that their presence leads others to contribute larger amounts and to contribute more frequently.

#### (ii) Work environment investment

Although public goods games provide helpful insights, their realism is constrained by the fact that in absolute terms players never lose anything. A rare study in a more realistic setting is provided by a large-scale analysis of the work rate of supermarket cashiers, wherein work rates can be accurately determined electronically through produce scan rates [68]. Individual variation in cashiers' work rates tended to be consistent and substantial, i.e. some cashiers worked considerably harder than others. Using natural introductions (arising from worker shift-changes), Mas & Moretti [68] found that the introduction of cashiers that were 10% more productive on average led to a 4% increase in work rates of slow-working cashiers. By contrast, hard-working cashiers did not reduce their work rate when paired with a slow worker. Thus, neither defection (individuals did not reduce work rates in the presence of others with low work rates) nor evidence of free-riding (cashiers increased, not decreased work rates with the introduction of a hard-working cashier) was found. Instead, there was cooperative matching or ‘positive spill over’. In economic terms, having the optimal mix of cashiers at any one time was estimated to produce the same output in 123 529 less hours worked per year, saving the company $2.5 million annually.

#### (iii) Warfare in small-scale societies

Small-scale societies suggest that certain key individuals may promote cooperation in many contexts including hunting, travel and inter-societal warfare [69]. We focus on the latter due to the abundance of ethnographic accounts. The most common pattern in small-scale warfare is for a group of warriors to engage in a surprise raid against members of another group where the goal is to injure or kill one or more victims [70]. Men are not compelled to join raiding parties and may drop out prior to the raid occurring as often happens. Warriors on a successful raid obtain personal benefits such as status or items of value, while all group members receive non-exclusive benefits such as deterrence and access to territory [71].

Quantitative studies indicate large individual differences in the frequency and/or intensity of participation in raiding parties across a broad range of ethnographic contexts. Among the Yanomamo of Venezuela, of 137 men who had participated in the death of another individual, 60% participated in only one killing while a small group of men participated in more than 10, with one individual participating in the death of 16 enemies [72]. Among the Waorani of lowland Ecuador, the vast majority of the raids included or were precipitated by two men before they were killed in revenge attacks [73]. Among the pastoralist Nyangatom of southwest Ethiopia, membership of small-scale raids is similarly variable with a small number of men participating in the majority of raids [74]. These studies reveal substantial inter-individual variation in contributions to a particular kind of cooperation in which potential costs and benefits are high.

These large differences in participation raise the question of whether high-contributing individuals tend to catalyse cooperation by others. Although ethnographic accounts of warfare are insufficiently detailed to answer this question definitively, the common presence of war leaders across diverse cultural contexts suggests that certain individuals may motivate participation by other group members. Although small-scale warfare occurs without chains of command or formal sanctions for defection, *ad hoc* or institutionalized leadership is a common characteristic (reviewed in [69]). War leaders generally mobilize other participants, develop tactics and possibly take more risk in conflict activities. In a salient example, Cheyenne war chiefs were expected to be killed in conflict [75]. Indeed, some of these war chiefs would loop a rope around themselves and attach it to a peg in the ground at the front line of the combat zone, where they would remain until other warriors in their group successfully forced the enemy back. Among the Kapauku of New Guinea, war leaders tend to lead attacks [76] and, for the Jie of Uganda, they sometimes go out alone in front of their allied warriors [77]. The apparent ubiquity of leaders cross-culturally, suggests they can function to motivate other individuals to contribute more to conflict.

## 3. Explaining variation in contributions

One problem with accounting for variation in individual contributions to a public good is that there can be multiple modes through which individuals can contribute. For example, contributions to rearing offspring can be manifest in provisioning, protection and defence, or thermoregulation. Measuring all contributions, let alone quantifying their respective costs and benefits represent a major challenge. Notwithstanding, parental contributions to offspring provisioning tend to be comparable in most bi-parental care settings but not in cooperative group settings. On the one hand, this is hardly surprising: bi-parental care systems comprise a breeding pair in which each member typically has similar fitness interests, while more cooperative groups comprise a variety of individuals whose current fitness costs and benefits of investment are often divergent. On the other hand, the variation observed in group cooperation scenarios is seldom predicted by traditional theory because prolonged investment asymmetries are expected to lead to attenuation or termination of investment by high contributors [4,6,10,18]. So, how can we explain the origin and persistence of substantially asymmetric contributions? Part of the explanation must lie with differing individual benefit and/or cost functions (figure 2).

### (a) Differing benefits

A number of benefits-based hypotheses can potentially account for variation in individual contributions in more cooperative settings. For example, and assuming comparable cost curves, those helping to rear first-order kin should work harder than those helping more distant relatives [9]. Similarly, those with more to gain from advertising their status, ability or quality, assuming contributions to be honest [20], or from living in large groups [21,78,79], should contribute more than those with less to gain. Finally, even where contributions to cooperation occur as a means of gaining experience [80], individuals are expected to vary their investment as a function of how much experience they need. Although not always subject to mathematical formulation, the attraction of many of these benefits hypotheses for explaining variation is that generally ‘investment-in’ equates to ‘fitness-out’. In other words, reducing investment in cooperation (e.g. in response to lazy co-investors) will normally be tantamount to reducing personal fitness, in which case it will be counter-selected. So differential personalized benefits of cooperation should select against defection [24,81]. In this regard, a priority for future work is to clarify the potentially divergent benefits on offer for each individual in a group. While such an approach is commonly adopted in cooperative breeding settings, it is less so in other contexts highlighted. For example, what are the benefits of investing heavily in a chimpanzee hunt, food gathering or inter-tribal warfare, given that the spoils are often shared with non-contributors? One obvious hypothesis is that the ‘spoils' are preferentially delivered to certain individuals. Key individuals or otherwise heavy investors might not only gain a greater share of the benefit, but could also gain other benefits, including social status and sexual partners [69,82–84]. If this idea is upheld more generally, it could mean that pressures to defect, and the tragedy of the commons phenomenon, are less pervasive factors than they are usually assumed to be.

While personalized benefits are undoubtedly important, they almost certainly cannot explain all individual variation in investment in cooperation. Indeed, past research indicates that individual differences in personalized benefits appear to exert only a weak influence on individual levels of cooperative contributions, at least in cooperative breeding settings. For example, relatedness asymmetries to the brood explain individual contributions to rearing offspring in some cooperative breeders (e.g. [44,85–87]), but not most [43]. Similarly, differential benefits arising from group augmentation or social prestige fail to explain variation in individual contributions in most cases tested [44,88–90]. Thus, despite isolated exceptions, so far variation in accruable benefits largely fails to explain the apparently universal variation in individual contributions to cooperation. An alternative lies with contrasting cost functions.

### (b) Differing costs

It is likely that the costs of investment vary between individuals in cooperative groups, for such groups usually comprise individuals that vary in age, condition, ability or outside options. In the chimpanzee impact hunter example described above, it is likely that impact hunters are particularly adept at hunting monkeys and suffer relatively reduced costs of doing so [61]. Additionally, in cooperative breeders, contributions are commonly associated with a broad range of cost-correlates (age, foraging success, body mass and rates of mass gain) and supplemental feeding experiments commonly generate increased contributions [25,26,38,39,78].

Such correlates of costs do not explain all the variation and in many cases, they also have little explanatory power. In meerkats (*S. suricatta*), individual contributions to cooperative activities are significantly influenced by age, sex and body condition as well as foraging success, rates of morning weight gain [78] and contributions to the previous breeding attempt [25]. However, after controlling for such effects, circulating levels of cortisol and/or prolactin explain significant variation in contributions, at least in male helpers [91,92]. In addition, still the most important determinant of individual contributions remained the identity of the individual itself [78,93]. There are two possible explanations for the sometimes surprisingly poor explanatory power of differential costs: either (i) multiple costs combine to account for the variation (as the meerkat example suggests); and/or (ii) we have yet to discover and measure the most salient costs. Measuring costs precisely might be more difficult if they are often manifested at the physiological level. There is a rich literature on the effects of early environmental effects on physiological measures of condition, including telomere lengths, the efficiency of insulin pathways, organ capacity and metabolism [94–98], but so far none of these have been analysed with respect to cooperative investment [23]. Nevertheless, it is also unlikely that variable cost functions, even if measured precisely, explain all the variation in all contexts. For example, it is difficult to envisage differential costs explaining most of the variation in individual contributions in the workplace or in public goods games, despite the fact that in both cases benefits are shared equally. And, it is difficult to see how key individuals, including war leaders, are able to absorb the costs sufficiently to explain their dramatically greater contribution.

### (c) Synthesis and the role of (epi)genetics

We should not be surprised if differential benefit and cost functions provide only part of the explanation given that individuals vary genetically and have varied development. Despite large pedigrees, especially in bi-parental care systems and cooperative breeders, quantitative genetic methods have rarely been applied to explain variation in investment in cooperation (e.g. [99]). For example, decisions to help are known to have a heritable genetic basis in western bluebirds (*Sialia mexicana*), although whether this also influences quantitative variation in levels of investment is not known [100]. Levels of cooperative investment may also have an epigenetic basis, as epigenetic affects are known to influence social behaviour [101] and expression of maternal care [102], but to our knowledge this is as yet unexplored. In addition, ‘personality’ characteristics which are thought to be influenced by early developmental effects on gene expression are known to influence cooperative tendencies in chimpanzees. For instance, Bullinger *et al.* [103] found that when captive chimpanzees were offered the chance to cooperate in obtaining equal amounts of food, they mostly did so (91% occasions), but three out of eight individuals consistently initiated while another three consistently joined, even though all individuals obtained the same reward. If personality traits like those found by Bullinger *et al*. have effects on hunting, they could account for the observed patterns in terms of proximate mechanism (see [104] for a discussion of the adaptive significance of personality traits).

An important outstanding question therefore is how genes and their expression influence cooperative contributions and how genes for extreme cooperation (or key individuals) can be maintained in a population despite associated costs. Two possibilities are worth considering: (i) key individuals gain substantial personal benefits, in addition to those benefits that are shared across all contributors; and (ii) under significant density-dependence, as all cooperative species probably experience, selection on alternative fitness-maximizing strategies leads to some individuals pursuing a high-risk, high-reward strategy.

In conclusion, individuals might vary in their contributions to cooperation if they stand to gain differential benefits or incur differential costs (figure 2). There is little question that these benefits and costs hypotheses provide some level of explanation for individual variation in contributions, although the degree to which each does so appears to vary among systems and circumstances. Critical evaluation of the benefits on offer at the level of each individual, along with rigorous investigations of associating costs, are challenging, but needed, directions for future research. Further, we suggest that serious consideration of epigenetic and genetic influences are now pressing.

## 4. When cooperation begets cooperation

If epigenetic or genetic influences account for significant variation in individual contributions to cooperation within groups, we need to be able account for the maintenance of genes for high investment within populations despite obvious counter-selection. While variation in individual-level cost–benefit functions must play some role (see above), we suggest that positive response rules could play an important facilitating role in maintaining genetic variation for variable contributions to cooperation. Here, we consider three hypotheses that might explain when cooperation begets cooperation. These include (i) information asymmetries; (ii) coercion and advertising in a biological market; and (iii) changing the shape of benefit functions or allowing individuals to shift their cost–benefit functions as a consequence of the actions of others.

### (a) Imperfect information concerning adaptive investment

The idea that imperfect information can generate positive (and negative) matching was proposed to account for the observations of positive matching of contributions in the experimental study on great tits (see above; [36]). Using an adaptation of the negotiation models of McNamara *et al.* [15], Johnstone & Hinde [27] formalized the idea mathematically. The critical findings were that individuals with more information should contribute more and that matching should arise when information regarding brood demand was imperfect such that it benefitted the lower investing, less informed individual, to use the other's provisioning rate as a cue of current brood demand. Support for this model has additionally been found in long-tailed tits. Males that experienced increases in contribution by their partners (induced experimentally by begging playbacks) responded by increasing their own contributions. This occurred during the early nestling period, which was a time when females have greater information than males about the hunger state condition of the nestlings due to their high brooding levels [105].

The value of the imperfect information hypothesis for explaining matching in other contexts has not yet been assessed. In any context, this hypothesis would predict that, all else being equal, those with more information will invest at a level closer to the optimum than those with less information. This means that increases in quality of information could lead an individual to invest at a lower rate or at a higher rate than others, depending on circumstance. Either way, those with less information should match those with more information.

While quality of information might play a role in any of the case studies presented above, it seems unlikely to play a primary role in explaining observations of positive responses. For example, why a few hard-working individuals in a cooperative group of ants, chimps or humans would be more informed than the mass of low-working individuals is unclear. Likewise, public goods players have the same level of information, but commonly vary in their contributions [66]. Finally, information was modelled and ruled out as being the explaining factor in the Mas & Moretti [68] supermarket study, because it was found to predict free-riding rather than the observed positive matching, and cannot explain the result that only those in direct view of the hard-working individuals raised their work rates. Thus, although asymmetries of information precision have been used to explain matching in some cases of bi-parental care, its utility remains to be assessed elsewhere.

### (b) Coercion and advertising in a biological market

In principal, coercion could influence individual contributions and responses to the contributions of others [29,106], but will often be counter-selected outside of a competitive market [23]. In the context of this paper, biological market theory proposes that cooperation is a form of competition, used to signal worth as a group member or future mate [30]. As such, it can predict both variation in contributions and positive matching of contributions: the former because those individuals of a given age or condition are selected to compete in the market, while younger individuals in poorer condition are not; the latter because competition in the market should elevate levels of contribution to the public good. For example, consider the case where individuals must help rear nestlings if they wish to remain on a territory (so-called pay-to-stay hypothesis [107]). If the number of individuals that benefit from being on territories exceeds the number of places, competition should arise, with one potential outcome being an escalation in cooperation. Similarly, if status in a group, population or society is linked with investment in the common good, and there is competition over status because high status individuals are preferred as social or sexual partners, then increasing investment by one should be met with increasing investment by another (e.g. [20]).

Both coercion and advertising hypotheses have attracted theoretical attention in both cooperative breeding and human cooperation, although generally under the guises of pay-to-stay and social prestige theory in the former [81], and in the latter, punishment, sanctions, policing [108] or indirect reciprocity with image scoring, respectively [81].

That both coercion and advertising can explain cooperative matching is intuitive, for they almost certainly represent viable explanations to understand cooperative matching in human settings. Indeed, both mechanisms have received some empirical support. For example, Mas & Moretti [68] suggested that the risk of social exclusion—arguably a form of punishment—was the greatest contributor to increased rates among lazy workers in the visual field of more arduous workers. Fear of sanctions has also been shown to promote contributions in public goods games [109], suggesting that the threat of punishment may play a key role in motivating cooperation. In addition to coercion, contributions to cooperation may serve as a means of advertising group norms or individual quality. For instance, in Weber and Muringham's study (see above; [67]), consistent contributors were thought to affect other players' contributions because they advertised cooperative norms. A recent study investigating online donations to charity showed that escalating contributions also occur in contexts in which individuals use contributions as a competitive signal [110]. Finally, as noted above, we would not be surprised if status benefits were on offer during warfare, hunting and food gathering in humans, and that such benefits lead others to join forces in the market. Together, these lines of evidence suggest that, at least in humans, individuals may use cooperation strategically in social interactions and that various strategies can lead to cooperative matching.

Although the coercion and ‘cooperating to compete’ appear relevant for accounting for at least some of the variation in responses to cooperative investment observed in humans, elevations in status or sanctions are unlikely to provide the only explanation for variation in cooperative investment. For instance, in public goods games, individuals vary in their contributions despite anonymity [66] and do not necessarily defect in the presence of free riders or punish those that contribute little [111]. In addition, the coercion and biological market hypotheses will struggle to account for most cases of cooperative matching in animals since evidence for pay-to-stay and social prestige are scant at best (see above, [81,88–90,112,113]). There are a number of explanations for this general lack of evidence, including: prohibitive costs of monitoring the contributions of others, the cognitive challenge of knowing when to punish ‘lazy’ individuals, or simply a lack of selection on cooperation as an advertisement. More generally, there is almost no firm evidence to suggest that lazy contributors can be forced to work harder through punishment, coercion, policing or sanctions, despite some suggestions [114]. This assertion should not be confused with the clear evidence that aggression can reduce reproduction in meerkats [115] or subordinate reproduction in social insects [116], and as a result, promote cooperation. Thus, while we do not rule out the obvious importance of coercive tactics in catalysing cooperative investments or the importance of biological markets, particularly in humans, it appears to lack general applicability in animals.

### (c) Changing benefit and/or cost functions

Traditional theoretical treatment of investment patterns in bi-parental and cooperative care systems generally make three assumptions. First, increasing investment is associated with diminishing benefit functions and accelerating cost functions (figure 1). Second, although individuals can have specific cost and benefit functions (figure 2), for each individual, the two functions approximate mirror images. Third, although the optimal level of investment by a given individual is sensitive to the contribution of co-investors, the shape of the benefit or cost function does not change with changing investment of others. Each assumption makes general sense in the context of bi-parental care, but one or more is likely to be violated in more cooperative settings.

### (d) Changing benefit functions

**Figure 3.**
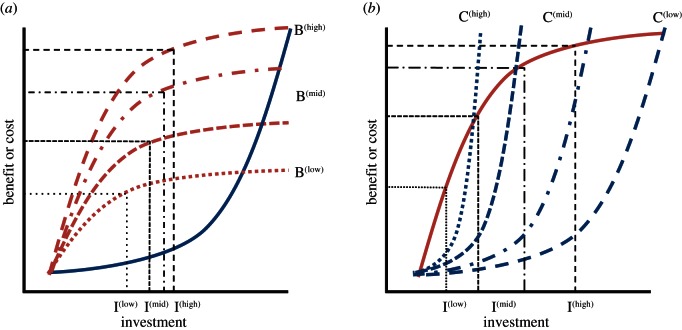
Consequences of shifting cost–benefit functions on cooperative contributions. (*a*) Consider a scenario in which all individuals have the same cost function (solid line), and where the benefit function for a given individual positively co-varies with the cooperative investments provided by others. It follows that if the benefits on offer are greater (B^(high)^ versus B^(low)^) in the presence of high investors, then individuals should also invest more heavily (I^(high)^ versus I^(low)^). Note that benefit functions might also be sigmoidal, generating a partly accelerating function (see [32] for ramifications). We have maintained the more classically assumed functions because we agree that in most cases the relevant parameter space of benefits will be linear-diminishing. (*b*) Similarly, consider a case where all individuals have the same benefit function (solid line), but where the costs of contributing vary as a function of the work rate of co-investors. For example, if the costs of contributing decline (C^(high)^ to C^(low)^) when in the presence of high investors, then individuals should change their investment from I^(low)^ towards I^(high)^. In both cases (*a*) and (*b*), positive relationships are expected between the contributions of co-investors. Note, in contrast to figure 1, in this case, the different benefit and cost curves in (*a*) and (*b*), respectively, pertain to the same individual depending on whether their co-investors are high, mid or low contributors. See text (§4*c*) for examples of how the shape of benefit and cost functions can vary as a function of co-investor contributions. Again, although cost functions might deviate from concave-up, we agree that such a shape is most general to the parameter space occupied by most individuals contributing to a cooperative venture. (Online version in colour.)

In more cooperative species, there are good reasons for supposing that the shape of benefits functions can vary as a function of the number, or levels of contribution, of co-investors (figure 3*a*). For example, if in larger groups individuals can contribute more effectively to multiple fitness-maximizing strategies (e.g. provisioning, defence, rearing higher quality offspring), then greater benefits might be available overall, leading to positive relationships among levels of investment by individuals within groups [48]. Similarly, in cooperative breeders, particularly eusocial insects, females commonly lay more, smaller eggs for increasing work forces [23]. One consequence is for larger groups to have greater accruable benefits than smaller groups. Where investments change linearly with helper number, we might expect helpers to maintain levels of contribution with increasing group size, but where they increase nonlinearly, we might expect positive responses [21,31,32,117]. In each case, the consequence of increasing benefits functions in the presence of helpful co-investors will be to maintain or increase (not decrease) individual investment levels with the addition of co-investors. This might also apply to public goods games in humans where the benefits curve is established to be a function of individual levels of investment. Here, the catalysing effects of ‘generous' contributors might be explained by altering benefits functions because the accruable benefits fundamentally increase as a function of the number of players, while the costs are individually paid.

We wish to make two key points here. First, although benefit functions must at some point diminish, because cooperative groups, particularly eusocial insects and microbes, can reach substantial group sizes during colony growth, individuals might more often operate around the linearly increasing (even accelerating) zone of the benefit function [32] than is typically assumed in bi-parental care models. Second, related, the actual shape of the benefits function might also be expected to change in at least some cooperative societies as a function of changing numbers of co-investors (e.g. figure 3*a*). More specific tests of these hypotheses are clearly required. Integral to such tests is a quantitative measure of: (i) how the relationship between the number of co-operators (or their level of investment) in a given situation relates to the benefits of offer for each individual; and (ii) how manipulation of cooperator number (or effort) affects individual contributions when constraints of diminishing benefits are experimentally relaxed. To our knowledge, few if any experiments have done both, which will include increasing the contributions of a select number of individuals as well as increasing the size of the accruable benefits on offer. The ‘synergy-type’ hypotheses outlined predicts that cooperative matching will arise where cooperative groups are operating on an accelerating part of the curve [32] or where the shape of the individual benefit curves are sensitive to the contributions of co-investors. We foresee such hypotheses being most common in eusocial insects and microbes, where benefit functions are highly flexible as a consequence of variation in colony size, or in public goods games, where the overall benefit on offer can be manipulated easily.

### (e) Changing cost functions

The case for changing cost functions is perhaps more intuitive and potentially more general. This is because all cooperative species tend to live in challenging niche environments; as a consequence, it follows that the individual cost function might be sensitive to the number (or investment) of other group members. For example, if cooperation leads to increased foraging efficiency or reduced predation risk, the individual costs of investing in the public good might also decline. Certainly, chimpanzee hunting of red colobus monkeys is rarely successful without the presence of active partners, perhaps because the costs of breaching monkey defences to reach the infant prey are maximal when there is only one hunter. Similarly, a lone warrior is unlikely to fare well against his rivals if he goes to battle alone. Finally, in most cooperative breeders, individuals probably benefit from group-living, and in at least some, the costs to a female or breeding pair of rearing offspring successfully can be very high [23,24].

Consider then, a case where the benefits function is of the classic diminishing form, but the cost function varies with numbers of co-investors or levels of co-investment (from extreme to classic (figure 3*b*)). Under such a scenario, a single individual can gain a small fraction of the total benefit on offer, before the costs of increasing investment are prohibitive [16]. Note that because the benefit is low and the costs are typically high, defection might often be the best strategy. Reaping maximal benefit will require ‘galvanizing’ a workforce, to reduce the costs to each individual. Generating a workforce can be achieved through coercion, if benefits tend to be personal and a single individual has more to gain than others, coalition formation, if the benefits are shared more equitably, or simply through coordination. Whichever way, the key point here is that if individual cost functions are sensitive to the investment of others, then increasing contributions of one should be positively matched by others.

To our knowledge, this hypothesis has never been tested experimentally, although the suggestion has been made with regards to cooperative hunting in chimpanzees [61]. In essence, the prediction is that removal or additions of group members, particularly key contributors, will generate reductions and enhancements to contributions of at least some group members, respectively. Both of these predictions counter those arising from bi-parental and current cooperative care models, which predict partial changes in the opposite direction (see above). However, a potential problem is that such experiments will also potentially change the benefits on offer, because remaining individuals can contribute to a greater or lesser share of a fixed public good. To remove this effect, the size of the public good needs to be reduced or increased to be proportional to that which was on offer pre-manipulation. Our prediction is that if cost functions vary with the number or contributions of others, then individual contributions will positively covary when the individual-level benefits on offer are maintained across the experiment.

### (f) Synthesis

Whether or not individual benefit or cost functions can be influenced favourably by the contributions of others has received almost no empirical attention to our knowledge. And, as a consequence, this has only recently begun to receive theoretical interest (e.g. [31,32,48,104,117]). We hypothesize here that contribution-mediated changes to individual cost–benefit functions offers a general means to explaining two of the critical phenomena that we highlight, namely: (i) positive matching of individual contributions; and (ii) the occurrence of key individuals. Rather intuitively, we expect that where the overall benefits are fixed within events, cooperative matching among group members will be governed by the positive effects of the presence or contributions of other group members on reducing each other's cost functions (figure 3*b*). For example, within a group breeding event, the size of the benefit on offer is fixed by offspring number (or potential quality), while within a group hunting event it is fixed by the size of the prey targeted—although in both cases benefits can vary among groups and events, leading to between-group or between-event variation in accruable benefits. By contrast, when the reward varies as a function of investment, as in public goods games, then we expect changing benefits functions to have a greater impact on cooperative matching (figure 3*a*). Finally, of course, changing both benefits and costs functions might be feasible. For example, in inter-tribal warfare, both the individual cost and benefit functions might vary favourably within the increasing contributions (or numbers) of others.

## 5. Conclusion

Individual contributions to cooperation vary dramatically across taxa and increases in either the number or contributions of investors can be associated with an unexpected increase in the contribution of other investors. Neither pattern is expected from classic theory on bi-parental and cooperative care (figure 1). Our primary aim using a broad, although selective, case study approach is to show that examples of each are neither limited nor exceptional. The relative paucity of the current evidence might stem in part from a lack of formal theoretical and empirical attention. First, we agree that variation in individual contributions will stem from among-individual variation in personal cost–benefit functions, coupled with stabilizing effects of personalized benefits, but urge greater consideration of epigenetic and genetic roles. Second, we hypothesize that cooperative matching arises, in part, when the shape of individual cost and benefit functions is sensitive to the number and/or contributions of others. More specifically, matching will be expected when an increase in investment by one either increases the accruable fitness available to others or reduces others' investment costs. Finally, the occurrence of key individuals might be explained by such a mechanism. Assuming such individuals accrue significant fitness, on average, their galvanization of support in others might arise from their specific ability to increase the benefits and/or reduce the costs of a given level of investment by others. Although we hope to inspire more formal theoretical attention of these ideas, we urge deeper interest by empiricists in using experimental approaches to understand variation in individual contributions and to determine individual-level responses to changes not only in the number of contributors but also in their levels of investment. By doing so, we will be able to gauge how commonly, and under what circumstances, cooperation begets cooperation.
